# Influence of Mindfulness on Levels of Impulsiveness, Moods and Pre-Competition Anxiety in Athletes of Different Sports

**DOI:** 10.3390/healthcare11060898

**Published:** 2023-03-21

**Authors:** Laura C. Sánchez-Sánchez, Clemente Franco, Alberto Amutio, Jaqueline García-Silva, Juan González-Hernández

**Affiliations:** 1 Department of Personality, Evaluation and Psychological Treatment, Faculty de Psychology, University of Granada, Campus Cartuja, 180071 Granada, Spain; 2 Department of Psychology, University of Almería, Carretera Sacramento, S/N, La Cañada de San Urbano, 04120 Almería, Spain; 3 Department of Social Psychology, University of the Basque Country (UPV/EHU), Barrio Sarriena, S/N, 48940 Leioa, Spain; 4 Facultad de Educación y Ciencias Sociales, Universidad Andres Bello, Santiago de Chile 7591538, Chile

**Keywords:** mindfulness, awareness, sport, psychosocial variables

## Abstract

Training in emotional regulation skills is one of the most important resources for the adaptation of athletes to contexts of sports pressure, especially during competitions. This study explored the effects of a mindfulness programme (Flow Meditation) on levels of impulsivity, mood and pre-competition anxiety-state in a sample of athletes (N = 41, 22.83 ± 5.62 years). Participants were randomly assigned to an intervention group (N = 21; 14 males and 7 females) which received the intervention over 10 weeks (a weekly session) and a control group (wait-list; N = 20; 13 males and 7 females). The variables under study were assessed through different questionnaires at pre- and post-test (T1–T2) in both groups. The mindfulness intervention was effective in reducing impulsivity (cognitive *(t* = −4.48, *p* ≤ 0.001, Cohen’s *d* = 1.40), both motor (*t* = −4.03, *p* ≤ 0.001, Cohen’s *d* = 1.20) and unplanned (*t* = −5.32, *p* ≤ 0.001, Cohen’s *d* = 1.66)), mood (tension (*t* = −4.40, *p* ≤ 0.001, Cohen’s *d* = 1.37), depression (*t* = −4.56, *p* ≤ 0.001, Cohen’s *d* = 1.42), anger (*t* = −7.80, *p* ≤ 0.001, Cohen’s *d* = 2.47), somatic anxiety (*t* = −5.28, *p* ≤ 0.001, Cohen’s *d* = 1.65), and cognitive anxiety (*t* = −6.62, *p* ≤ 0.001, Cohen’s *d* = 2.07) in the intervention group compared to the control group and with large to very large effect sizes. Mindfulness is a factor that enhances athletes’ ability to cope with high sport pressure and the healthy management of competition (e.g., fear of failure), or with their daily life.

## 1. Introduction

Physical exercise and sport have a positive effect on physical and mental health and on personal development [1]. However, in competitive sport, athletes must fulfil high expectations and be physically and mentally prepared to achieve peak performance. Today, more and more sport professionals recognise the importance of mental preparation to optimise performance and maintain mental health [2]. The relevance of psychological functioning to an athlete’s performance is reflected both in sporting outcomes and in the athlete’s well-being and mood. Within sports, there are a number of psychological traits or behaviours (e.g., impulsivity, anxiety, negative moods, or fatigue) that have been associated with deficits in emotional regulation skills and can negatively influence performance [3,4]. On the other hand, positive emotions and moods, such as joy and vigour, may contribute to increased self-confidence and sport performance [5,6].

From a cognitive–behavioural perspective, impulsivity could be defined as the tendency to behave without a sufficient process of the observation, analysis, and/or evaluation of consequences, in the form of tendencies towards risk-taking behaviour, rapid decision making, and lack of planning [7]. Impulsivity is generally considered a predisposition to perform quick and thoughtless actions in response to internal and/or external stimuli despite negative consequences for oneself and/or others. It is described in terms of the process involved: motor (i.e., action without thought), perceptual–attentional (i.e., lack of concentration on the task), and lack of planning or transcendence (i.e., prioritising when, how, and where the action occurs without giving value to the consequences for the future) [8,9,10]. In sport contexts, impulsive responding has been related both to the pursuit of greater and faster efforts to be perfect [11], fear of failure [12], and even self-destruction [13], and to negative emotions such as anger, aggressive behaviours, concentration difficulties, and poor performance [14,15].

As for anxiety, although scientific evidence has widely demonstrated that anxiety negatively affects psychosocial resources and individual conditions for sport performance [16,17], some athletes are able to perform under great pressure, while others drown under the combined weight of intense competition and high levels of physiological arousal and psychological stress. Anticipatory fear makes the individual more vulnerable [18], altering the functionality and adaptive capacities in terms of emotional [19,20], cognitive [21], and behavioural [22] responses linked to sport action, thus impacting on mood states and exacerbating activation processes [23,24]. Models such as the Multidimensional Theory of Competitive Anxiety identify the components of anxiety related to sport contexts [25], namely, cognitive anxiety (negative thoughts, expectations, and/or self-talk related to the competitive event), somatic anxiety (affective and physiological elements that directly affect the central nervous system), and self-confidence (related to the level of confidence and the perception of being prepared for the competition).

Understanding how the components of the impulsive and anxious response and mood states work also allows intervention on the psychological risks associated with sport situations and actions [26]. Early learning and/or training in psychological resources that promote emotional and behavioural regulation reduce discomfort in athletes (e.g., anger-anxiety, depression, fatigue, or withdrawal), mainly when they need their performance to adapt adequately and fluently to competitive situations or sporting demands [16,27], positively influencing sporting performance.

A growing number of studies have demonstrated the efficacy of psychological interventions, including mindfulness, on emotional and behavioural regulation in sports [28,29,30,31], hence the relevance of teaching regulatory skills, especially to young athletes participating in competitions [11,32,33], as they are subjected to greater sporting pressures (e.g., the obligation to win or to prove themselves to be the best).

In the 20 years since the incorporation of mindfulness in the context of sports, empirical findings have revealed effective outcomes associated with performance and personal well-being, as well as supporting the theorised mechanisms of change. Mindfulness is a form of meditation that involves intentional and non-judgmental awareness of the present moment, including physical sensations and experienced affective states [34,35]. The use of mindfulness facilitates directing one’s attention towards coping with the pressure coming from technical, physical, biological, and even professional aspects. However, although the main objective of mindfulness is to learn to accept the internal events (i.e., thoughts, emotions, etc.) involved in any activity in the present, in studies with elite athletes, it has been observed that their coping with negative thoughts improved and their sports performance was also enhanced [36,37,38].

Currently, we have several mindfulness interventions or programmes applied to the field of sport, including the Mindfulness–Acceptance–Commitment Approach (MAC) [38], Mindful Sport Performance Enhancement (MSPE) [39], Mindful Performance Enhancement, Awareness, and Knowledge (mPEAK) [40], Mindfulness Meditation Training for Sport (MMTS) [41], Berlin Mindfulness-Based Training for Athletes (BAT) [42], the Mindfulness–Acceptance–Insight–Commitment program (MAIC) [43], among others [44]. In Spain, the Flow Meditation programme (Flow Meditation) [45] has been applied in those with different disorders and different populations, facilitating the reduction in impulsivity and aggressiveness in students and of anxiety and depression levels in older adults [46,47].

Expanding the repertoire of mindfulness strategies available to athletes and health professionals is a useful and relevant topic in this field. Furthermore, mindfulness interventions in the sport domain have mainly focused on variables such as coping, concentration levels, and relaxation [44]. Nowadays, trainers and sport professionals have no doubt that possessing good physical skills does not guarantee optimal sport performance, so they have started to take the psychological skills and personality characteristics of players seriously (i.e., anxiety and/or anger management, feelings of depression and fatigue, resilience, and even self-efficacy) [5,6].

The present study is the first to include a number of psychological variables that so far have barely been studied in the field of mindfulness applications to sport, but which are critical for improving well-being and sport performance. Thus, the aim of this study was to explore the efficacy of a mindfulness programme called Flow Meditation [45] on impulsivity, mood, and pre-competition anxiety in a group of athletes. On the basis of the scientific literature mentioned above, the starting hypothesis postulates that the intervention will improve impulsive response, mood (i.e., depression, tension, anger, fatigue, and vigour), and pre-competition anxiety in the intervention group compared to the control group.

## 2. Materials and Methods

### 2.1. Participants

The sample of the present study consisted of 41 federated athletes who participated in official competitions at the provincial, regional, or national level and who were studying different degrees at the University of Almeria (Spain). The ages of the participants ranged from 18 to 32 years (22.83 ± 5.62 years). In total, 6% of the participants were involved in athletics, 8% in tennis, 13% in swimming, 14% in basketball, 16% in handball, 21% in volleyball, and 22% in football. The intervention group consisted of 21 participants (14 males and 7 females), (21.74 ± 4.93 years), while the control group consisted of 20 athletes (13 males and 7 females) (23.92 ± 6.31 years). The sample was randomly assigned to one or the other group, controlling the variables of gender and sporting activity, so that there was a similar number of men and women and athletes from the different sporting disciplines in both groups, thus avoiding the interference of these variables in the results of the study. Informed consent was obtained from all subjects involved in the study.

### 2.2. Instruments

- Impulsivity. The Spanish adaptation of Oquendo et al. [48] of the Barratt Impulsivity Scale (BIS-11) [49,50] was applied. This questionnaire is composed of 30 items that are grouped into 3 subscales of impulsivity: (i) Cognitive impulsivity (8 items): tendency to make quick decisions; (ii) motor impulsivity (10 items): propensity to act solely on the stimulus of the moment, without thinking about the consequences; and (iii) impulsivity due to lack of planning (12 items): indicates the lack of planning of future actions. Each item consists of 4 Likert-type response options (never or almost never, sometimes, quite often, and always or almost always). The internal consistency of the different scales, obtained using Cronbach’s alpha coefficient, showed values of 0.78 for the cognitive impulsivity subscale, 0.75 for motor impulsivity, and 0.66 for impulsivity due to non-planning.
- Mood. The original reduced version of the Profile of Mood States (POMS) [51], validated in Spanish by Fuentes et al. [52], was used. The abbreviated form of the Profile of Mood States consists of a list of 29 adjectives assessing five mood states: tension (6 items), depression (6 items), anger (6 items), vigour (6 items), and fatigue (5 items). The items are rated on a five-point Likert-type scale, from 0 = not at all; 1 = a little; 2 = moderately; 3 = quite a lot; to 4 = very much. The extent to which each of the moods described in each adjective has been experienced during the last week, including the day on which the questionnaire is answered, is assessed. Cronbach’s alpha values for the reduced versions showed the following scores: tension (0.64), depression (0.69), anger (0.61), vigour (0.72), and fatigue (0.66).
- Competitive anxiety. The Spanish version of the Competitive Anxiety Inventory CSAI-2R by Andrade et al. [53] was administered in athletes. It consists of 18 items distributed in 3 dimensions: (i) somatic anxiety (8 items), which represents the perception of bodily indicators of anxiety, such as muscle tension, increased heart rate, sweating, and stomach discomfort; (ii) cognitive anxiety (5 items), which encompasses the negative feelings that the subject has about their performance and the consequences of the possible outcome; and (iii) self-confidence (5 items), which refers to the degree of confidence that the athlete believes they have about their chances of success. A Likert-type response format of four alternatives from 1 “not at all” to 4 “very much” was used. Internal reliability showed adequate consistency with Cronbach’s alphas of 0.91 for the somatic anxiety dimension, 0.71 for the cognitive anxiety dimension, and 0.65 for the self-confidence dimension.

### 2.3. Design and Procedure

A quasi-experimental design was carried out with a wait-list control group, with intra- and inter-group comparisons. 

Firstly, we proceeded to obtain the study sample by convenience sampling, for which we offered a course entitled *“The development of mindfulness in sport”*, aimed at athletes enrolled at the University of Almeria who were participating in official competitions at the provincial, regional, or national level. Once the study sample (n = 41) was constituted, we started to obtain the pre-test measurements for the dimensions of the variables of impulsivity, mood states, and anxiety-state, for which all the participants enrolled in the course were provided with questionnaires for the evaluation of these variables to be completed individually, on the weekend before the start of the intervention programme. Once this pre-test score was obtained, the participants were randomly assigned to the control and intervention groups, controlling the variables of gender and sporting discipline, so that they would not interfere with the results of the study. In the same week, the intervention programme began to be applied to the intervention group, with one session per week for 10 consecutive weeks. The subjects in the control group (wait-list; n = 20) were informed that for reasons of space they would receive the course in a second turn. During the weekend after the end of the course in the intervention group, the levels of the different dimensions of the impulsivity, mood and anxiety variables were re-evaluated in both groups under the same conditions as in the pre-test phase.

### 2.4. Intervention Programme

The programme was applied in the intervention group over ten sessions, with a weekly session lasting one and a half hours each. The sessions were held in the evening in a soundproofed university classroom with a good room temperature and the necessary equipment for the meditations (e.g., movable chairs, mats, blankets). This intervention programme consisted of learning and daily practice for 40 min of a meditation technique for the training and development of mindfulness called Flow Meditation [45,54]. Participants were given basic instructions on how to meditate at home: to always choose the same time and place (preferably a quiet place, with little light and good temperature); to allow at least two hours after eating before meditating and not to carry it out at a time when they had to do something urgent afterwards; to choose a comfortable posture, with the back upright; not to look for any special result, but to abandon themselves to whatever arises during meditation, flowing and without expecting big changes at the beginning, since meditating is a habit that takes time to be acquired and the effects take their time to develop and be noticed. The main objective is not to try to control thoughts, sensations, or feelings, nor to modify or change these for another but, on the contrary, to leave them free, accepting any private event that may appear or arise spontaneously in our consciousness. In other words, during practice, one becomes aware of the presence of thoughts without analysing their content or veracity, but rather developing the awareness that thoughts (as well as sensations) change every moment and that they are constantly flowing. Therefore, during the practice of this mindfulness technique, participants will understand experientially that thoughts arise and disappear continuously and that they are subject to a continuous flow, learning, in this way, to be present, open, and balanced before any phenomenon or mental or emotional process that occurs in their minds.

In each of the 10 sessions, in addition to the learning and practice of mindfulness, various metaphors and exercises from Acceptance and Commitment Therapy [55,56] as well as stories from the Zen tradition [57] and from Vipassana meditation [58] were employed. The aim of these stories and exercises was to reinforce the idea that when we try to control or eliminate the upsetting and unpleasant thoughts, emotions, and bodily sensations we experience, they become chronic, thus aggravating the psychological discomfort. So, the best option is therefore to become aware of them, accept them as they appear, and let them flow freely. Other components of the mindfulness Flow Meditation programme were body scan exercises [34]. After the research was completed, the mindfulness course was conducted with the control group, as indicated at the beginning of the study. The intervention programme was developed by an instructor with extensive experience in both practising and teaching mindfulness meditation techniques.

### 2.5. Data Analysis

Data were analysed with the statistical software SPSS software—IBM SPSS Statistics for Windows, Version 25.0. Armonk, NY, USA: IBM Corp.

First, Shapiro’s normality test was conducted. Since the variables conformed to the normal distribution, parametric tests were then performed. Levenne’s test was applied to corroborate the homogeneity of variances. Second, Student’s *t*-tests for independent samples were performed to test whether there were significant differences between the intervention group and the control group before starting the intervention. Third, intra-group measures (*t*-tests for dependent samples) were performed between pre-test or time 1 (hereafter T1) and post-test or time 2 (hereafter T2). Fourth, post-treatment measures were compared between groups (*t*-tests for independent samples) to verify the extent to which these changes were statistically significant and on which variables. For variables where statistically significant differences were found, the effect size was calculated using Cohen’s *d*. The interpretation is as follows: 0.2 is considered a small effect, 0.6 a moderate effect, 1.2 a high effect, and 2.0 a very high effect. Finally, a multiple linear regression analysis was performed with the variable self-confidence at T2 as the dependent variable, due to the divergence found between intra-group and inter-group results on this variable. In addition, descriptive analyses were performed in both groups for all variables at T1 and T2.

## 3. Results

### 3.1. Descriptive Data

The descriptive data for both groups for all variables at T1 and T2 are presented in Table 1. Student’s *t*-tests for independent samples found no significant differences between the main variables between the groups before the intervention (*p* < 0.05).

### 3.2. Intra-Group Comparison (T1–T2)

A comparison of the changes between T1 and T2 measures in both groups was made. As shown in Table 1, there were statistically significant changes in the intervention group in the following variables: the three subscales of the BIS-11, cognitive impulsivity, motor impulsivity and impulsivity due to non-planning; the POMS, in tension, depression, and anger; and the CSAI-2R, in somatic anxiety, cognitive anxiety, and self-confidence. In all of them, these statistically significant changes meant an improvement in the intervention group. In this group, the effect size was in all cases between high and very high. However, in the control group, statistically significant changes only occurred in the somatic anxiety and cognitive anxiety scales of the CSAI-2R, but these changes meant a worsening, i.e., an increase in anxiety levels. In this group, the effect size for those significant changes was moderate (see Table 1).

### 3.3. Inter-Group Comparisons

Differences between the two groups were also calculated on T2 measures, and statistically significant differences were found in favour of the intervention group on the following variables: on the three subscales of the BIS-11, cognitive impulsivity, motor impulsivity, and impulsivity due to non-planning; on the POMS, on tension, depression, and anger; and the CSAI-2R, on somatic anxiety and cognitive anxiety. In terms of effect size, all these results ranged from high (1.2) to very high (2.47) (see Table 2).

### 3.4. Multiple Linear Regression Analysis

Given that in the intra-group analysis, statistically significant differences were found in the self-confidence variable at T2 in the intervention group, but no statistically significant differences were found in the inter-group analyses, a multiple linear regression analysis (Table 3) was performed with self-confidence as the dependent variable, in order to discern which variables were the best predictors of its values. The independent variables were group (intervention versus control group), gender, type of sport played, and the score on the same variable at T1. The results reveal that the two most predictive variables were, in first place, the score in self-confidence at T1, followed by the group to which they belonged.

## 4. Discussion

The results of the application of the Flow Meditation intervention programme in the sample of athletes studied reveal an improvement in the intervention group in cognitive impulsivity, motor impulsivity, and impulsivity due to lack of planning. There was also an improvement in tension, depression, and anger, as well as in somatic anxiety, cognitive anxiety, and self-confidence, confirming the hypotheses formulated. In contrast, the control group showed an increase in somatic and cognitive anxiety.

Along the same lines, other studies have observed improvements in burnout [59], decreased stress and anxiety [3,37], improved mood [17], as well as higher levels of flow and improved sports performance [39,60,61,62] following the application of a mindfulness programme in athletes. Recent research suggests a reduction in depressive symptoms and an increase in psychological well-being [59,63] and self-compassion [3].

Mindfulness intervention with Flow Meditation can be indirectly considered a type of psychological training to optimise performance. Moreover, it is an effective strategy to improve concentration and emotion regulation in athletes. According to the systematic review on mindfulness intervention programmes by Bühlmayer et al. [64], mindfulness practice affects cognitive processes and is considered increasingly significant within sport psychology training. The effects of increased mindfulness promote greater concentration, awareness, and acceptance, which are necessary to counteract the negative effects of stressful sport performance [65,66].

In addition to performance anxiety and negative thoughts, the negative effects of competition include fatigue, boredom, and pain [65]. Mindfulness interventions do not focus on directly altering or changing dysfunctional thoughts and emotions, but rather the athlete’s relationship with their physiological and psychological states. However, collateral changes occur, such as improved cognitive skills [67] and even increased pain tolerance in injured athletes. Thus, Mohammed et al. [68] indicate a notable decrease in stress and an improvement in mental health after the application of the MBSR programme (Mindfulness-Based Stress Reduction). Similarly, other authors report effects on reducing the risk of injury and even facilitating recovery from serious injuries [59,69]. Other studies suggest that even brief mindfulness training can prevent deterioration in athletic performance and produce psychological benefits [3,60].

The results obtained indicate an increase in the variable of self-confidence in the intervention group. Recent research supports the findings of this study, suggesting that mindfulness is closely related to this variable, which is essential for promoting sports performance [70,71,72,73], thus preventing the onset of mental disorders and improving quality of life [38]. In short, it is possible to conclude that mindfulness training enhances cognitive and emotional resources for managing highly demanding situations and, at the same time, will contribute to correcting maladaptive behaviours of athletes in competition.

The mechanisms involved, which can act both directly and indirectly, include: neurobiological changes, the increased emotional regulation and reduction in negative emotions, such as anxiety and anger [74,75], increased attentional control [67,76,77], flow [78,79], reduced rumination and irrational beliefs (e.g., perfectionism), reduced worry and self-judgment [3,44], decreased experiential avoidance [80], increased positive emotions and self-efficacy [6,81], and even (self-)compassion [3,24].

Flow Meditation constitutes a Second-Generation Mindfulness programme, and it includes ethical, existential, and spiritual aspects. Thus, the aim is not only to improve sports performance and individual well-being, but also to promote mindfulness skills for everyday life and values such as equanimity and transcendence [35,45]. This objective comes along with new lines of future research proposed by some authors [44,82]. In this way, Kee et al. [44] suggest that mindfulness training in athletes should incorporate the Taoist concept of *wu-wei,* whereby athletes learn to set aside and transcend ideas of winning or losing while playing and concentrate solely on the task, without judging their actions and paying attention only to those external stimuli relevant to the task. In Western terms, the more related word might be flow (i.e., to let go of effort, to flow, or to go with the flow). The flow state has been defined as an optimal state of awareness in which one is totally absorbed and connected with what one is doing, even to the exclusion of all thought or emotion, and in which mind and body work together harmoniously, pleasantly, almost automatically, and without conscious effort. Experiencing this state is associated with a positive impact on self-perceived sport competence and sports performance [79,83]. In this sense, optimal performance does not require the volitional control of internal states [38].

Athletes trained from a young age, for whom mindfulness is already part of their daily lifestyle, develop what has been called trait mindfulness, rather than state mindfulness, which is limited to specific situations or novice meditators [29]. Both describe mindfulness about the task in the present moment and, when they occur together, constitute consolidated processes of acceptance and engagement with the task, without value judgments, promoting achievement and the processes to accomplish it [84]. Moreover, the application of mindfulness-based therapies, in which present-moment awareness is enhanced, seems to have a more positive influence on psychological variables related to competition than other therapies involving an altered state of consciousness, such as hypnosis [85].

Finally, the application of this type of treatment in athletes could also function as a protective factor against other problems linked to competition, such as disorders related to body image or eating behaviour [86].

The study described here has several limitations that need to be noted: (i) the small number of participants belonging to each sport activity; (ii) the absence of an active control group; (iii) the exclusive use of self-report measures; (iv) the absence of a follow-up phase of the treatment effects.

Future studies should continue to evaluate the efficacy of mindfulness in controlled trials and in larger populations in order to generalise the findings and confirm the effects of the intervention on different psychological variables that affect the mental health and performance of athletes. Finally, it is of utmost relevance to assess the long-term effects on the population studied.

## Figures and Tables

**Table 1:** Intra-group comparison of T1–T2 measures in the intervention and control groups with *t*-tests for dependent samples of T1–T2 measures in both groups and effect sizes.

Scales Subscales	Intervention Group				Control Group					
	Mean (SD) T1	Mean (SD) T2	*t*	*p*	Effect Size (Cohen’s *d*)	Mean (SD) T1	Mean (SD) T2	*t*	*p*	Effect Size (Cohen’s *d*)
BIS-11										
Cognitive Impulsivity	20.62 (3.88)	17.62 (2.13)	5.86	<0.001	1.81	21 (3.18)	21.35 (3.13)	−0.41	0.687	_____
Motor Impulsivity	20.76 (3.66)	18 (2.12)	6.50	<0.001	2.0	21.1 (3.48)	21.75 (3.67)	−0.49	0.631	_____
Unplanned Impulsivity	21.76 (3.63)	19.43 (2.6)	4.72	<0.001	1.46	23.45 (3.3)	23.95 (2.84)	−0.52	0.609	_____
POMS										
Tension	15.86 (2.31)	12.86 (2.48)	9.49	<0.001	2.93	16.15 (2.03)	15.95 (1.99)	0.37	0.718	_____
Depression	12.62 (2.66)	10.14 (1.82)	7.55	<0.001	2.33	12.15 (1.95)	12.85 (1.98)	−1.61	0.125	_____
Anger	10.43 (1.63)	8.19 (1.17)	6.93	<0.001	2.14	11.1 (1.52)	11.7 (1.66)	−2.04	0.055	_____
Vigour	18.95 (1.16)	19.1 (0.94)	−0.47	0642	_____	18.6 (1.18)	18.85 (1.27)	−0.61	0.549	_____
Fatigue	8.95 (1.24)	8.38 (1.07)	1.39	0180	_____	8.45 (1.36)	8.7 (1.08))	−0.68	0.506	_____
CSAI-2R										
Somatic Anxiety	21.1 (2.84)	17.48 (2.91)	9.83	<0.001	3.03	21.95 (3.93)	22.7 (3.42)	−2.52	0.021	0.80
Cognitive Anxiety	14.1 (1.92)	11.1 (1.61)	12.55	<0.001	3.87	14.00 (1.41)	14.55 (1.73)	−2.15	0.045	0.68
Self Confidence	14.1 (1.3)	14.81 (1.17)	−3.1	0.006	0.96	14.9 (1.83)	14.65 (1.88)	1.23	0.234	_____

Degrees of freedom, intervention group = 20; control group = 19.

**Table 2:** Independent sample *t*-tests for T2 measures in the intervention and control groups and effect sizes.

Scale	Subscales	*t*	df	Effect Size (Cohen’s *d*)	*p*
	Cognitive Impulsivity T2	−4.48	39	1.40	<0.001
	Motor Impulsivity T2	−4.03	39	1.2	<0.001
	Unplanned Impulsivity T2	−5.32	39	1.66	<0.001
	Tension T2	−4.4	39	1.37	<0.001
	Depression T2	−4.56	39	1.42	<0.001
	Anger T2	−7.8	33.98	2.47	<0.001
	Vigour T2	0.71	39	_____	0485
	Fatigue T2	−0.95	39	_____	0348
	Somatic Anxiety T2	−5.28	39	1.65	<0.001
	Cognitive Anxiety T2	−6.62	39	2.07	<0.001
	Self-confidence T2	0.33	31.57	_____	0.747

df (degrees of freedom).

**Table 3:** Linear regression of factor associated with self-confidence in T2.

Variables	β	SE	Standard β	*t*	*p*	95.0% Confidence Interval for β	
						Lower Limit	Upper Limit
Group	−0.801	0.307	−0.264	−2.612	0.013	−1.422	−0.179
Gender	−0.353	0.318	−0.111	−1.113	0.273	−0.998	0.291
Type of Sport	−0.002	0.079	−0.003	−0.028	0.978	−0.163	0.159
Self-confidence T1	0.805	0.102	0.847	7.865	0.000	0.597	1.012

## Data Availability

Data are available on request from the authors.
